# Deeper Than the Metabolite: A Novel Genetic Mutation in an Indian Child With Glutaric Aciduria Type 1

**DOI:** 10.7759/cureus.97016

**Published:** 2025-11-16

**Authors:** Preeti Srivastava, Sumeet S Biswal, Ratan Kumar, Shikha Swaroop, Sanjay K Tanti

**Affiliations:** 1 Pediatrics, Tata Main Hospital, Jamshedpur, IND; 2 Pediatrics, Manipal Tata Medical College, Manipal Academy of Higher Education (MAHE), Jamshedpur, IND

**Keywords:** gcdh gene, glutaric aciduria type 1, metabolic disorders, novel genetic mutation, organic acidemia

## Abstract

Glutaric aciduria type 1 (GA-1) is a rare autosomal recessive metabolic disorder caused by the deficient activity of the mitochondrial enzyme glutaryl-CoA dehydrogenase (GCDH). This leads to the accumulation of neurotoxic metabolites, resulting in basal ganglia injury and neurologic dysfunction. We report a case of a one-and-a-half-year-old Indian male with GA-1 harboring a novel homozygous missense variant in exon 11 of the GCDH gene. The report highlights diagnostic challenges, the significance of genetic testing, and the effect of early intervention in these patients. Prompt recognition remains essential to prevent irreversible neurological sequelae.

## Introduction

Glutaric aciduria type 1 (GA-1) is a rare autosomal recessive inborn error of metabolism (IEM), categorized within the group of organic acidemias. The condition results from a deficiency of the mitochondrial enzyme glutaryl-CoA dehydrogenase (GCDH) due to pathogenic variants in the GCDH gene [1]. The enzyme deficiency leads to accumulation of glutaric acid, 3-hydroxyglutaric acid, and glutaconic acid in body fluids [2], producing neurotoxic effects-particularly on the basal ganglia, which may result in irreversible dystonia and developmental delay if untreated [3]. The diagnosis is established using tandem mass spectrometry (TMS) to detect elevated C5‑dicarboxylic acylcarnitines and gas chromatography‑mass spectrometry (GC‑MS) of urine organic acids [1,4]. Genetic testing via next-generation sequencing (NGS) confirms the diagnosis and enables family counseling [5]. We discuss the case of a child with GA-1 harboring a novel GCDH mutation, highlighting clinical presentation, diagnostic challenges, and implications for genetic counseling.

## Case presentation

**Figure 1:**
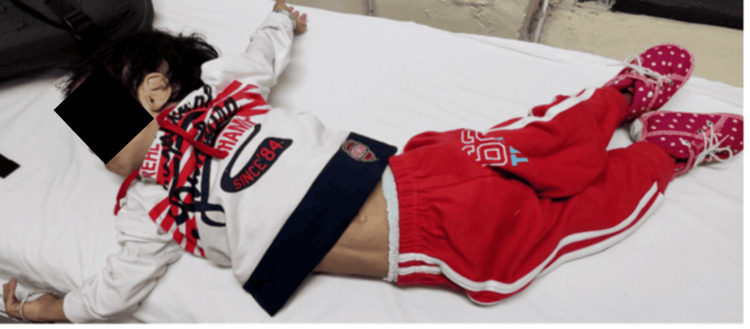
Photo of the patient demonstrating dystonia and opisthotonic posturing

A 1.5-year-old boy, born to non-consanguineous parents with an unremarkable antenatal and perinatal history, presented with multiple generalized tonic-clonic seizures involving all four limbs and up-rolling of the eyeballs. He had also been experiencing poor neck control, dystonia, and involuntary movements for the past two months, along with a fever for the past week. He was receiving levetiracetam, clobazam, clonazepam, baclofen, and trihexyphenidyl, with partial control of symptoms. Developmentally, he had achieved milestones corresponding to a developmental age of eight to nine months: sitting without support, immature pincer grasp, and use of bisyllables without meaningful words. There was a lack of eye contact and regression of milestones - loss of neck holding and crawling - after one year of age. His younger sibling was healthy, and there was no similar family history. Neurological examination revealed dystonia (Figure 1), macrocephaly, and a positive Babinski reflex.

**Figure 2:**
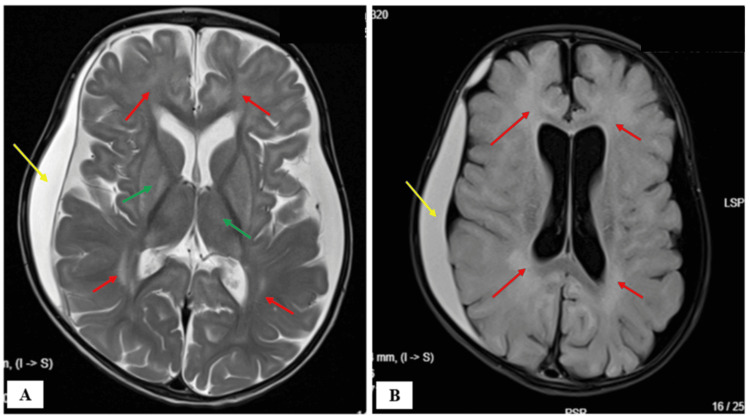
Brain MRI findings (a) Axial T2 and (b) FLAIR images show hyperintensity in the subcortical white matter of the bilateral cerebral hemisphere, more predominant in the frontal, temporal, and parietal lobes of the brain (red arrows in A and B) and bilateral basal ganglia (green arrows in A). There is a subdural hemorrhage subjacent to the right cerebral hemisphere (yellow arrows in A and B) MRI: magnetic resonance imaging; FLAIR: Fluid-attenuated inversion recovery

Cerebrospinal fluid (CSF) analysis and electroencephalogram (EEG) were normal. Brain MRI (Figure 2) showed bilaterally symmetrical hyperintensities on T2‑FLAIR with diffusion restriction on DWI involving subcortical white matter of the cerebral hemispheres, particularly frontotemporal regions, along with bilateral basal ganglia, thalami, substantia nigra, and central tegmental tracts. Delayed myelination and a large subacute subdural hemorrhage adjacent to the cerebral hemispheres, causing mass effect, were noted. These findings were suggestive of glutaric aciduria.

TMS findings were within normal limits, while GC‑MS of urine showed marked elevation of glutaric acid and 3‑hydroxyglutaric acid. Whole‑exome sequencing (WES) identified a homozygous missense variant (Table 1) in exon 11 of the GCDH gene (chr19: g.12897789G>T; Depth: 274×). The p.Gly390Val variant was absent in 1000 Genomes and gnomAD (v3.1, v2) databases, indicating novelty. Although classified as a variant of uncertain significance (VUS), it correlated clinically.

**Table 1:** Whole exome sequencing result: variant of uncertain significance related to the given phenotype was detected

Gene (transcript)	Location	Variant	Zygosity	Disease (OMIM)	Inheritance	Classification
GCDH (+) [ENST00000222214.10]	Exon 11	C1169G>T [p.Gly390Val]	Homozygous	Glutaricaciduria type 1 (OMIM#231670)	Autosomal recessive	Uncertain significance (PM2, PP3)

The child was advised a low‑lysine diet and carnitine supplementation along with symptomatic treatment. However, due to non‑compliance, the disease progressed, and the child succumbed by the age of 3½ years.

## Discussion

Organic acidemias are a group of IEM resulting from deficiencies of enzymes responsible for amino acid degradation, leading to the accumulation of abnormal and toxic organic acids. GA‑1, a cerebral organic acidemia, results from a deficiency of GCDH, impairing the catabolism of lysine, hydroxylysine, and tryptophan. The resultant accumulation of glutaric acid and derivatives leads to neurotoxicity. Organic acidemias can be broadly classified in three groups: (a) branched‑chain acidemias, which includes methylmalonic acidemia, propionic acidemia, isovaleric acidemia, 3‑methylcrotonylglycinuria, and 3‑methylglutaconic aciduria; (b) multiple carboxylase deficiency, which includes holocarboxylase synthetase deficiency and biotinidase deficiency; and (c) cerebral acidemias, which includes GA-1, aspartoacylase deficiency (Canavan disease), and 4‑hydroxybutyric aciduria [6,7]. The clinical spectrum of GA‑1 varies from asymptomatic neonates to severely affected individuals who experience neurological crises following metabolic stress.

Two primary phenotypes of GA-1 are recognized. The first is infantile-onset GA-1, in which the first encephalopathic crisis occurs before six years of age (commonly between three months and three years). Of note, 80-90% of untreated children experience such crises, 95% within the first two years of life. These crises often follow febrile illness, vaccination, or fasting, leading to bilateral striatal injury and progressive dystonia. Subdural hemorrhages may appear spontaneously or after mild head trauma [1,2]. The second phenotype is late-onset GA-1,* *in which the first crisis occurs after six years of age; such patients may present with chronic neurologic findings, including headaches, macrocephaly, epilepsy, tremor, dementia, or peripheral neuropathy [6].

Our patient’s phenotype and imaging were consistent with infantile‑onset GA‑1. MRI findings of basal ganglia hyperintensity and cortical involvement corroborated biochemical results. Genetic analysis revealed a novel homozygous missense variant p.Gly390Val in exon 11 of GCDH, corresponding clinically despite its VUS classification. The identification of novel variants reinforces the necessity of genetic assessment in suspected GA‑1 to supplement biochemical and radiological findings [8]. Early diagnosis and diet‑based therapy considerably improve outcomes. Delays lead to irreversible neurological impairment, including dystonia and cognitive decline. This case broadens the known mutational spectrum of GCDH and demonstrates the importance of genetic studies in elucidating GA‑1 heterogeneity. Genetic confirmation further assists family counseling and carrier screening, enabling informed reproductive decisions.

Limitations

This report is limited by the absence of extended follow‑up data and incomplete treatment compliance. Additionally, genotype-phenotype associations for new GCDH mutations remain insufficiently studied. Further research should focus on innovative therapeutic approaches such as enzyme replacement or gene‑directed therapies to address underlying metabolic abnormalities. Expanding variant databases will also refine genotype‑phenotype correlation and improve predictive diagnostic accuracy.

## Conclusions

This report underscores the importance of combining biochemical analysis, neuroimaging, and genetic testing when evaluating suspected GA-1. Prompt diagnosis and intervention remain crucial to prevent neurological deterioration. Identification of the novel GCDH mutation p.Gly390Val contributes valuable insight into the expanding genetic diversity of GA‑1. Ongoing clinical vigilance, database expansion, and longitudinal research will further enhance our understanding and management of this rare condition.
